# Activation of Immune and Defense Responses in the Intestinal Mucosa by Outer Membrane Vesicles of Commensal and Probiotic *Escherichia coli* Strains

**DOI:** 10.3389/fmicb.2016.00705

**Published:** 2016-05-11

**Authors:** María José Fábrega, Laura Aguilera, Rosa Giménez, Encarna Varela, María Alexandra Cañas, María Antolín, Josefa Badía, Laura Baldomà

**Affiliations:** ^1^Departament de Bioquímica i Biologia Molecular, Facultat de Farmàcia, Institut de Biomedicina de la Universitat de Barcelona, Universitat de BarcelonaBarcelona, Spain; ^2^Department of Gastroenterology, Digestive System Research Unit, Institut de Recerca Vall d’Hebron, CIBER EHD, Instituto de Salud Carlos III, University Hospital Vall d’Hebron, Universitat Autònoma de BarcelonaBarcelona, Spain

**Keywords:** Nissle 1917, probiotics, gut microbes, membrane vesicles, microbiota-host crosstalk, intestinal mucosa, Caco-2/PBMC co-culture

## Abstract

The influence of microbiota in human health is well-known. Imbalances in microbiome structure have been linked to several diseases. Modulation of microbiota composition through probiotic therapy is an attempt to harness the beneficial effects of commensal microbiota. Although, there is wide knowledge of the responses induced by gut microbiota, the microbial factors that mediate these effects are not well-known. Gram-negative bacteria release outer membrane vesicles (OMVs) as a secretion mechanism of microbial factors, which have an important role in intercellular communication. Here, we investigated whether OMVs from the probiotic *Escherichia coli* strain Nissle 1917 (EcN) or the commensal *E. coli* strain ECOR12 trigger immune responses in various cellular models: (i) peripheral blood mononuclear cells (PBMCs) as a model of intestinal barrier disruption, (ii) apical stimulation of Caco-2/PMBCs co-culture as a model of intact intestinal mucosa, and (iii) colonic mucosa explants as an *ex vivo* model. Stimulations with bacterial lysates were also performed. Whereas, both OMVs and lysates activated expression and secretion of several cytokines and chemokines in PBMCs, only OMVs induced basolateral secretion and mRNA upregulation of these mediators in the co-culture model. We provide evidence that OMVs are internalized in polarized Caco-2 cells. The activated epithelial cells elicit a response in the underlying immunocompetent cells. The OMVs effects were corroborated in the *ex vivo* model. This experimental study shows that OMVs are an effective strategy used by beneficial gut bacteria to communicate with and modulate host responses, activating signaling events through the intestinal epithelial barrier.

## Introduction

Intestinal microbiota has a great impact on human health. These microbial populations provide crucial benefits to the host, such as metabolic activities that promote energy harvest and nutrient absorption from food, development of the host immune system, and prevention of gut colonization and infection by pathogens (O’Hara and Shanahan, 2006; Garrett et al., 2010; Krishnan et al., 2015). Disturbances in microbiota composition can contribute to the development of diverse pathologies (Belkaid and Hand, 2014). Recent metagenomics studies have revealed certain microbial profiles (dysbiosis) associated with inflammatory, metabolic or infectious diseases (Le Chatelier et al., 2013; Robles-Alonso and Guarner, 2013; Sears and Garrett, 2014). Manipulation of gut microbiota through diet, probiotics and other approaches is a promising therapeutic strategy to prevent or alleviate disorders correlated with imbalances in the intestinal microbiota (Shanahan, 2011; Foxx-Orenstein and Chey, 2012).

The intestinal ecosystem is characterized by dynamic and reciprocal interactions between the microbiota, the epithelium and the mucosal immune system. This process requires elaborated regulatory mechanisms to ensure symbiosis and prevent aberrant responses that lead to pathological states. The epithelial layer is the first line of defense against pathogens and is also the surface where the host interacts with the microbiota. Epithelial cells have a critical role in sensing intestinal bacteria. They are equipped with a great variety of receptors that can recognize specific conserved molecular patterns in microbes pattern-recognition receptors (PRRs), such as the membrane-bound toll-like receptors (TLRs). TLRs mainly signal through the adaptor protein MyD88 to activate master transcription factors like NF-kappa B, thus triggering cytokine secretion and activation of host defense mechanisms. Other signaling PRRs are the cytosolic NOD-like receptors, which can modulate apoptosis and inflammatory responses (Jacobs and Braun, 2014; Thaiss et al., 2014; Caballero and Pamer, 2015). However, the gut microbiota does not directly interact with the intestinal epithelium. Both cell types are physically separated by the mucus layer. Commensal microbiota resides in the outer mucus layer, whereas the inner mucin layer is highly compacted and prevents bacteria from accessing the epithelial cells (Johansson et al., 2008; Vaishnava et al., 2011). In this scenario, a key issue is how the crosstalk between microbiota and the host is established. Proteins or factors secreted by microbiota have a relevant role in intestinal communication as they can diffuse through the mucin layer and come into contact with the intestinal mucosa cells.

All bacteria release extracellular vesicles as a means of communication with the environment. The best characterized are the outer membrane vesicles (OMVs) produced by Gram-negative bacteria. These vesicles act as a bacterial secretion pathway for selected proteins and other active compounds in a protected environment. In addition, they allow cell–cell communication, without direct intercellular contact (Kulp and Kuehn, 2010; Kim et al., 2015). Many studies conducted in the last decade with Gram-negative pathogens showed that OMVs are internalized in the host cell and contribute to virulence by delivering cytotoxic factors, as well as mediators that interfere with the immune system (Ellis and Kuehn, 2010; Chatterjee and Chaudhuri, 2013; Schertzer and Whiteley, 2013). At present, microbiota vesicles are seen as key players in signaling processes in the intestinal mucosa (Kaparakis-Liaskos and Ferrero, 2015; Olsen and Amano, 2015). However, studies in this field are still very scarce and mainly focused on *Bacteroides fragilis* (Shen et al., 2012; Stentz et al., 2015), a main Gram-negative group in the gastrointestinal tract of mammals. OMVs from this commensal promote immunomodulatory effects and prevent experimental colitis in mice. These effects are mediated by the capsular polysaccharide A (PSA) through TLR-2. However, a transcriptomic analysis in dendritic cells stimulated with *B. fragilis* ΔPSA-OMVs revealed changes in gene expression that are PSA-independent (Shen et al., 2012). *Akkermansia muciniphila* OMVs are also able to protect the progression of experimental-induced colitis in mice (Kang et al., 2013). Regarding Gram-positive bacteria, studies performed with *Bifidobacterium bifidum* LMG13195 showed that membrane vesicles from this probiotic can activate the maturation of dendritic cells, which trigger the regulatory T cells (Treg) response (López et al., 2012).

Our group reported the first vesicular proteome of a probiotic strain, namely *Escherichia coli* Nissle 1917 (EcN) (Aguilera et al., 2014). This proteomic study identified 192 vesicular proteins that have functions related to adhesion, immune modulation or bacterial survival in host niches, thus indicating that probiotic-derived OMVs contain proteins that can target these vesicles to the host and mediate their beneficial effects on intestinal function (Aguilera et al., 2014). EcN is a Gram-negative probiotic used in the treatment of intestinal disorders, especially in the maintenance of ulcerative colitis (Kruis et al., 2004; Chibbar and Dieleman, 2015). The strain, which was originally isolated from a soldier who survived a severe outbreak of diarrhea during the First World War, is a good colonizer of the human gut and positively affects gastrointestinal homeostasis and microbiota balance. It is known that EcN modulates the expression of antimicrobial peptides and the immune system function in the gut by expressing bacterial factors that specifically interact with host TLRs (Hafez et al., 2010).

The EcN-mediated effects have been evidenced from a great variety of *in vitro* and *in vivo* experiments performed essentially with live probiotic suspensions (Sturm et al., 2005; Schlee et al., 2007; Ukena et al., 2007; Zyrek et al., 2007; Arribas et al., 2009). The aim of this study was to define whether the immune modulation effects promoted by EcN are mediated through released OMVs. We extended the analysis to other commensal *E. coli* strains without probiotic traits, such as ECOR12. We analyzed the modulation of the cytokine/chemokine response by epithelial and immune cells upon stimulation with OMVs isolated from these strains in different *in vitro* and *ex vivo* models. We provide evidence that OMVs are internalized in polarized Caco-2 cells, and that these activated cells trigger an immune response in the underlying immunocompetent cells. *Ex vivo* experiments performed with colonic mucosa explants confirmed the role of OMVs in microbiota-gut signaling processes.

## Materials and Methods

### Bacterial Strains and Growth Conditions

The probiotic strain EcN (serotype O6:K5:H1) was provided by Ardeypharm (GmbH, Herdecke, Germany). ECOR12 is a commensal *E. coli* strain isolated from a healthy human stool sample (Ochman and Selander, 1984). Bacterial cells were grown at 37°C in Luria-Bertani broth (LB). Growth was monitored by measuring the optical density at 600 nm.

### Preparation of Bacterial Cell Lysates and OMVs

Bacterial lysates were prepared from cells grown in LB medium. Bacteria were collected by centrifugation (5,000 *× g*, 10 min, 4°C) and resuspended in phosphate buffer saline (PBS) for sonic disruption on ice. Cell debris was removed by centrifugation at 16,000 *× g* for 30 min at 4°C and the supernatant filtered through a 0.22 μm-pore-size filter to remove any residual cell.

Outer membrane vesicles were isolated from culture supernatants as described previously (Aguilera et al., 2014). In brief, bacterial cells grown overnight in LB were pelleted by centrifugation (10,000 × *g*, 20 min, 4°C); the supernatants were filtered through a 0.22 μm-pore-size filter (Millipore), concentrated by centrifugation in a Centricon Plus-70 filter device (Millipore) followed by an additional filtration step. Vesicles were then collected by centrifugation at 150,000 × *g* for 1 h at 4°C, washed and resuspended in PBS. Isolated OMVs were examined by transmission electron microscopy after negative staining as described previously (Egea et al., 2007).

Samples, either lysates or OMVs, were stored at -20°C until use. Sterility of samples was assessed on LB plates. Protein concentration was determined by the method of Lowry et al. (1951).

### Immunoblotting of Lipopolysaccharide (LPS)

Protein samples (bacterial lysates and OMVs) were separated on 15% SDS-PAGE and transferred to a Hybond-P polyvinylidene difluoride membrane by using a Bio-Rad MiniTransblot apparatus. The membrane was blocked in PBS-0.05% Tween-20 and 5% skimmed milk (blocking solution) for 1 h at room temperature, and then incubated with specific antibodies against LPS (Abcam; 1:5,000 dilution in blocking solution) for 16 h at 4°C. The secondary antibody was donkey anti-rabbit immunoglobulin horseradish peroxidase-linked, diluted 1:15,000 in blocking solution. The protein-antibody complex was visualized by using the ECL Plus Western blotting detection system (Amersham Pharmacia Biotech).

### Cell Lines and Growth Conditions

Caco-2 human colon adenocarcinoma cells (ATCC HTB-37) were obtained from the American Type Culture Collection. Cells (passages 55–65) were routinely grown at 37°C in a 5% CO_2_ atmosphere in Dulbecco’s modified eagle medium (DMEM) High Glucose containing 25 mM HEPES, 1% non-essential amino acids, 10% heat inactivated fetal calf serum (FCS) (Gibco BRL), penicillin G (100 U/ml) and streptomycin (100 μg/ml; Gibco BRL).

For Transwell cultures, 5 × 10^5^ Caco-2 cells were seeded on the apical compartment of 12 mm polycarbonate inserts (0.4 μm, Transwell Corning) and experiments were performed when confluent monolayers were fully polarized (18–20 days post-confluence). During growth and differentiation the medium was changed every 2 days in both compartments. Monolayer integrity was controlled by measurement of the transepithelial electrical resistance with a Millicell-ERS-2 volt-ohmmeter (Millipore, Madrid, Spain) and by visual assessment of cell layer integrity under the microscope. Prior to apical stimulation with OMVs or bacterial lysates, the medium was changed to DMEM High Glucose containing 25 mM HEPES, 1% non-essential amino acids, 1% heat inactivated FCS and gentamycin (150 μg/ml). For co-culture experiments, human peripheral blood mononuclear cells (PBMCs) were added to the basolateral compartment at a cell density of 2 × 10^6^ cells/ml.

### PBMCs Isolation

Peripheral blood mononuclear cells were isolated from fresh buffy coats of six healthy donors, provided by the “Banc de Sang i Teixits” of Barcelona according to the signed agreement with the Institution. Briefly, the buffy coat was centrifuged over a Hystopaque density gradient (Hystopaque-1077, Sigma Aldrich) following the manufacturer’s instructions. PBMCs were suspended in DMEM High Glucose containing 25 mM HEPES, non-essential amino acids, 1% heat inactivated FCS and gentamycin (150 μg/ml) and adjusted to 2 × 10^6^ cells/ml. Stimulation with OMVs or bacterial lysates was performed in 24 well plates (PBMCs only) or in 12 mm Transwell inserts (Caco-2/PBMCs co-culture).

### Organ Culture of Human Colonic Mucosa

Macroscopically normal colonic tissue was obtained after adenocarcinoma surgery from six patients undergoing right colon resection. Full-thickness colonic wall specimens distant from the tumor and without macroscopic lesions were rinsed under a saline jet and washed twice in sterile saline at 4°C. Mucosal samples weighing 25–35 mg each were separated from underlying tissue and placed with the epithelial surface facing up on culture filter plates (15-mm diameter wells with 500-μm bottom mesh, Netwell culture systems, Costar). Filters were suspended over wells containing 1.5 ml of medium RPMI 1640 (Life Technologies) supplemented with 2 mM glutamine and 150 μg/ml gentamicin. Tissues were incubated during 5 h at 37°C in humidified atmosphere and stimulated by the addition of OMVs or bacterial lysates, as described below. During this time oxygen supply was provided to preserve tissue integrity (95% O_2_/5% CO_2_). Tissue viability was assessed by measuring lactate dehydrogenase as described elsewhere (Borruel et al., 2003).

### Stimulation Conditions

To investigate the interaction between OMVs and cells of the intestinal mucosa, three different experimental approaches were performed: (i) direct stimulation of PBMCs (2 × 10^6^ cells/ml), (ii) apical stimulation of differentiated Caco-2/PBMCs co-cultures and (iii) apical stimulation of colonic mucosa explants. Each experiment was conducted six times (six individual donors in duplicate). Stimulations were performed with OMVs (50 μg/ml) or bacterial lysates (100 ng/ml) from EcN or ECOR12 strains. The concentration of these bacterial samples was selected according to experimental procedures reported elsewhere (Schaar et al., 2011; Güttsches et al., 2012). Cells were incubated at 37°C in a 5% CO_2_ atmosphere for 5 h (for expression analysis by RT-qPCR) or 24 h (for quantification of secreted cytokines). Incubation in growth medium was performed in parallel as a control. For RNA isolation the medium was removed and cells or tissue explants were suspended in appropriate volume of *RNA later^®^* (Ambion) to preserve RNA integrity and kept at -80°C until RNA extraction. For cytokine quantification, culture supernatants were collected from direct stimulated PBMCs or from the basolateral compartment in co-culture experiments. Supernatants were clarified by centrifugation (300 × *g*, 5 min) and stored in aliquots at -80°C until use. In the *ex vivo* experiments, both culture supernatant and tissue explants were collected and processed after 5 h stimulation.

### Cytokine and Chemokine Quantification in Culture Supernatants

Secreted IL-10, TNF-α, and MIP1α were measured using a cytometric bead array system (CBA FlexSet, BD Biosciences) according to manufacturer’s instructions and analyzed by flow cytometry (Gallios Beckman Coulter) in the Scientific and Technological Services of the University of Barcelona (CCiT – UB). Measurement of IL-8 and IL-6 was performed using ELISA sets (BD Biosciences). IL-22 was quantified by ELISA using the R&D System Duo Set.

### RNA Isolation and Quantitative Reverse Transcription PCR (RT-qPCR)

Total RNA was extracted from PBMCs and Caco-2 cells by using the Illustra RNAspin Mini kit (GE Healthcare) according to the manufacturer’s instructions. RNeasy Mini kit (Qiagen) was used to extract total RNA from colonic tissue samples. The concentration and purity of RNA samples were assessed by the ratio of absorbance at 260 and 280 nm in a NanoDrop^®^ spectrophotometer. RNA integrity was verified by visualization of 28S and 18S rRNAs after 1% agarose/formaldehyde gel electrophoresis.

RNA (1 μg for colon explants or 350 ng for PBMCs and Caco-2 cells) was reverse transcribed using the High Capacity cDNA Reverse Transcription kit (Applied Biosystems) in a final volume of 20 μl following manufacturer’s recommendations. RT-qPCR reactions were performed in a StepOne Plus PCR cycler (Applied Biosystems) by using the Taqman Gene Expression Master Mix, and the Taqman probes and primers for human IL-10, MIP1α, TNF-α, IL-6, IL-8, IL-12, IL-22, TGF-β, β-defensin-2 (hBD-2), β-defensin-1 (hBD-1) and mucin-1 (MUC1). The standard PCR program used was: one denaturation cycle for 10 min at 95°C followed by 40 cycles of 15 s at 95°C and 1 min at 60°C. A control reaction was performed in the absence of RNA. The housekeeping gene β-actin was used as a normalizing gene. Relative gene expression was calculated as fold-change compared with control and calculated by means of ΔΔCt formula.

### OMVs Labeling and Internalization Assay

To monitor OMVs internalization in intestinal epithelial cells, vesicles were fluorescently labeled with rhodamine isothiocyanate B-R18 (Molecular Probes) as described elsewhere (Bomberger et al., 2009). OMVs purified as described above were washed with PBS, resuspended in labeling buffer (50 mM Na_2_CO_3_, 100 mM NaCl, pH 9.2) in the presence of 1 mg/ml rhodamine isothiocyanate B-R18 and incubated for 1 h at 25°C. Labeled OMVs were pelleted by centrifugation at 100,000 × *g* for 1 h at 4°C, resuspended in PBS (0.2 M NaCl) and washed twice to fully remove the unbound dye. After a final centrifugation step, the rhodamine labeled-OMVs were resuspended in PBS (0.2 M NaCl) containing a protease inhibitor cocktail (Complete Protease Inhibitor Tablet, Roche) and stored at 4°C for up to 6 weeks.

OMVs internalization assays were performed using polarized Caco-2 cells (18–20 days post-confluence) grown in 96-well plate (Corning Incorporated, Costar^®^). Prior to the assay, the medium was replaced with rhodamine B-R18-labeled OMVs (2 μg/well) suspended in DMEM medium in the absence of phenol red and FCS. Cells were incubated at 37°C and fluorescence was measured over time using a Modulus^TM^ Microplate fluorescence (Turner BioSystems; Ex 570 nm; Em 595 nm). Fluorescence intensity was normalized for fluorescence detected by labeled-OMVs in the absence of epithelial cells.

OMVs internalization was assessed by confocal fluorescence microscopy. Caco-2 cells were grown in 8-well chamber slider (ibidi) and incubated with rhodamine B-R18-labeled OMVs (2 μg) at 37°C for 1 h, and then washed with PBS. Cells were fixed with 3% paraformaldehyde, permeabilized with 0.05% saponin (Sigma Aldrich) and blocked using PBS containing 1% bovine serum albumin. Nuclei were labeled with DAPI. To visualize cell boundaries, the peripheral zonula occludens ZO-1 protein was stained using anti-ZO-1 rabbit IgG antibody (Invitrogen) and Alexa Fluor 488-conjugated goat anti-rabbit IgG (Invitrogen). Confocal microscopy was carried out using a Leica TCS SP5 laser scanning confocal spectral microscope with 63x oil immersion objective lens. Images were captured with a Nikon color camera (16 bit). Fluorescence was recorded at 405 nm (blue; DAPI), 488 nm (green; Alexa Fluor 488), and 546 nm (red; rhodamine isothiocynate B-R18). Z-stack images were taken at 0.5 μm. Images were analyzed using Fiji image processing package.

### Statistical Analysis

Statistical analysis was performed using SPSS version 20.0 software (SPSS, Inc.) and data were expressed as mean ± SEM (*n* = 6). To establish the distribution of the data, the test Kolmogorov–Smirnov was run. To assess the effect of every experimental condition compared to the other conditions, the *t*-test (normal distribution) or the Mann–Whitney *U*-test (non-normal distribution) were performed. Differences between more than two groups were assessed using one-way ANOVA followed by Tukey’s test. Significant differences were established at *p* ≤ 0.05.

### Ethical Approval

All the procedures involving human patients were approved by the local ethics committees (Comité Ético de Investigación Clínica, Hospital Vall d’Hebron and Comité Ético del Banc de Sang i Teixits de Barcelona). Written informed consent was obtained from all patients (CEIC: PR(AG)56/2010).

## Results

### Comparative Protein Profile and LPS Analysis of OMVs Isolated from the Probiotic EcN and the Commensal ECOR12 Strains

**FIGURE 1:**
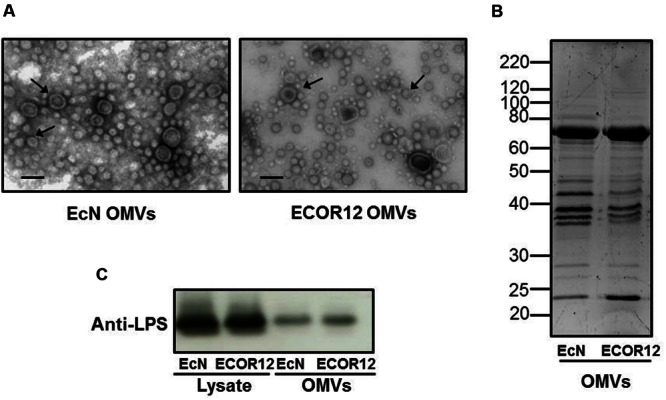
**Analysis of EcN and ECOR12 OMVs. (A)** Negative staining electron microscopy of isolated vesicles. OMVs are indicated by arrowheads. Bar = 50 nm. **(B)** Comparison of the protein profile of EcN and ECOR12 OMVs. Isolated vesicles (10 μg) were separated in a 10%-SDS-PAGE gel and stained with Sypro^®^ Ruby. Molecular size markers are indicated. **(C)** Western blot analysis of LPS in OMVs (0.1 μg protein) or in bacterial lysates (10 μg protein) obtained from the indicated strains.

Outer membrane vesicles from EcN and ECOR12 were isolated from culture supernatants. Examination of OMVs by transmission electron microscopy revealed that both strains release vesicles ranging from 20 to 60 nm in diameter (**Figure 1A**). To further compare the OMVs isolated from these strains, their protein profile was analyzed by SDS-PAGE and the LPS content was estimated by immunoblotting. Results showed similar protein profiles and LPS amount in both strain vesicles (**Figures 1B,C**). Immunoblotting of the LPS was also carried out in EcN and ECOR12 lysates (**Figure 1C**).

### OMVs from EcN and ECOR12 Induce Cytokine Secretion in PBMCs

Human PBMCs, which include several types of immunogenic cells, were used to evaluate the immunomodulatory effects of OMVs secreted by EcN and ECOR12 strains. Direct stimulation of these immune cells can be used as an *in vitro* model of intestinal inflammation and barrier disruption.

**FIGURE 2:**
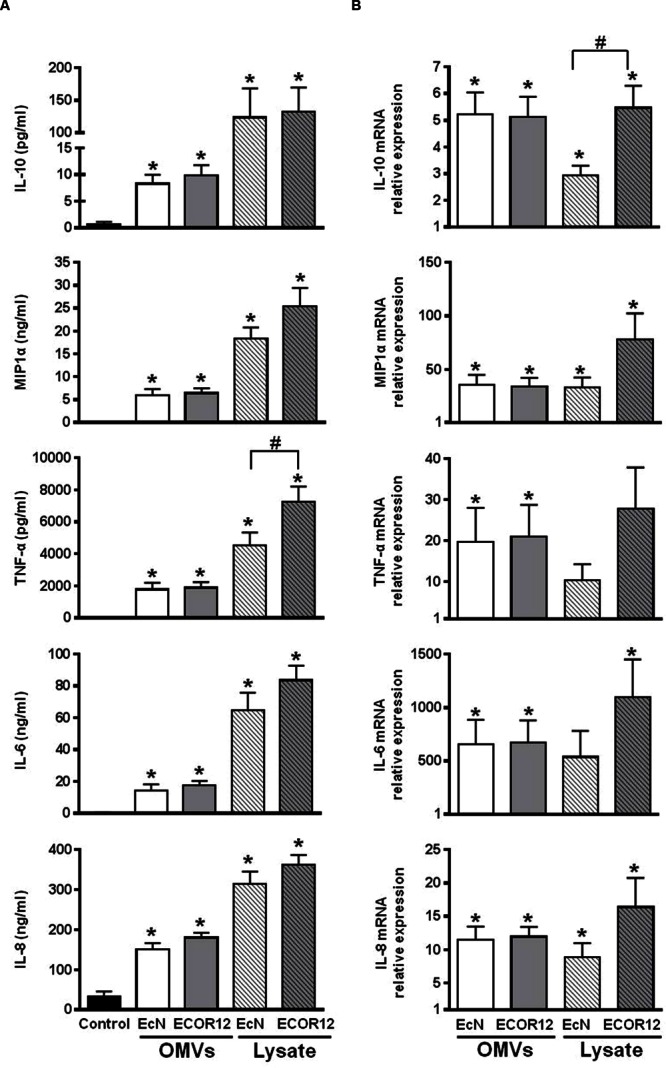
**Expression analyses of secreted cytokines and chemokines in PBMCs stimulated with OMVs or bacterial lysates.** PBMCs were challenged by addition of OMVs or bacterial lysates from EcN or ECOR12 strains. **(A)** Cytokine concentration in culture supernatants after 24 h stimulation. Data are expressed as mean ± SEM. **(B)** Relative gene expression levels in cells after 5 h stimulation. Data are presented as fold-change compared to untreated control cells. Statistical differences were assessed by one-way ANOVA followed by Tukey’s test. ^∗^*p* ≤ 0.05, versus control cells; #*p* ≤ 0.05, between cells treated with OMVs from EcN or ECOR12.

PMBCs were stimulated by the addition of OMVs for 24 h in the presence of gentamicin. Stimulations with bacterial lysates were conducted in parallel for comparison. As illustrated in **Figure 2A**, OMVs and lysates from both strains induced the secretion of IL-10, MIP1α, TNF-α, IL-6, and IL-8 by PBMCs (*p* ≤ 0.05). Secretion levels of MIP1α, IL-6, and IL-8 were in the ng range. Lysates triggered greater activation than OMVs in this cell model for all the cytokines and chemokines studied. ECOR12 lysates tended to promote increased secretion levels of proinflammatory cytokines than EcN lysates, although only the results for TNF-α were statistically significant (**Figure 2A**).

Expression of these inflammatory and immunomodulatory mediators was also analyzed by RT-qPCR after 5 h-stimulation. The results confirmed that all genes were upregulated in stimulated versus control cells (**Figure 2B**). Although, the changes in gene expression triggered by OMVs or lysates did not exactly match the secretion pattern of the soluble mediators released (**Figure 2A**), the tendency of ECOR12 lysates to produce greater cytokine activation was also apparent.

### OMVs from EcN and ECOR12 Activate Cytokine Production by PBMCs in Co-culture with Caco-2 Cells

To evaluate the crosstalk between OMVs, intestinal epithelial cells and immune cells, we used the Caco-2/PBMCs co-culture Transwell model that simulates the interaction of microbiota with the intestinal mucosa (Haller et al., 2000; Fang et al., 2010; Pozo-Rubio et al., 2011). In this model, signaling between epithelial and immune cells occurs through the release of soluble mediators.

**FIGURE 3:**
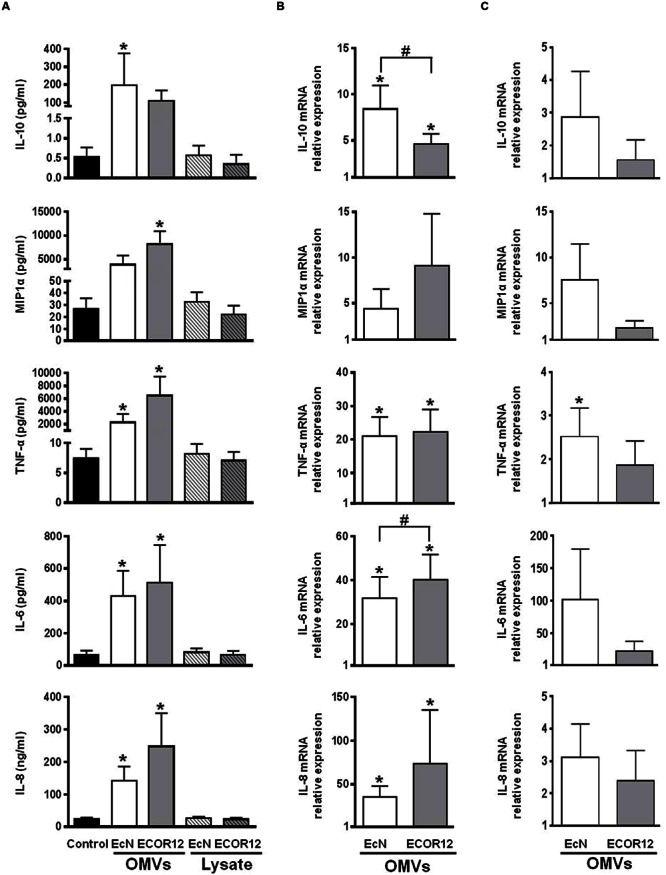
**Expression analysis of secreted cytokines and chemokines in Caco2/PBMCs co-cultures apical stimulation.** The apical surface of Caco-2 monolayers was challenged by addition of OMVs or bacterial lysates from EcN or ECOR12 strains. **(A)** Cytokine concentration in culture supernatants after 24 h stimulation. Data are expressed as mean ± SEM. Relative gene expression levels in Caco-2 cells **(B)** and in PBMCs **(C)** after 5 h stimulation with OMVs. Data are presented as fold-change compared to untreated control cells. Statistical differences were assessed by the *t*-test or by the non-parametric Mann–Whitney *U*-test. ^∗^*p* ≤ 0.05, versus control cells; #*p* ≤ 0.05, between cells treated with OMVs from EcN or ECOR12.

Differentiated Caco-2 cells in the apical compartment were challenged with OMVs from EcN or ECOR12, and with bacterial lysates for comparison. After 24 h incubation, the level of released cytokines was measured in the basolateral compartment. Transepithelial electrical resistance was monitored before and after each experiment to ensure the intact barrier function of the monolayer. The results presented in **Figure 3A** show that apical stimulation with OMVs elicited increased secretion of IL-10, MIP1α, TNF-α, IL-6 and IL-8, whereas lysates from the same strains did not produce any activation effect, yielding comparable secreted levels as untreated control cells. Stimulations of Caco-2 monolayers without underlying PBMCs were performed in parallel. Analysis of cytokine secretion in the basolateral compartment showed that polarized Caco-2 cells are almost unresponsive in the absence of crosstalk with immune cells (data not shown). This finding is in accordance with results reported by other groups (Zoumpopoulou et al., 2009; Fang et al., 2010; Pozo-Rubio et al., 2011).

In Caco-2/PBMCs co-cultures the cytokine secretion profile was clearly different from that of PBMCs directly exposed to bacterial samples. As the Caco-2 monolayer constitutes a physical barrier to the bacterial factors, these results showed that OMVs, but not lysates, could stimulate epithelial cells, which in turn may signal to the underlying PBMCs. In this co-culture model, activation of Caco-2 cells by OMVs was corroborated by RT-qPCR analysis of the expression profile of IL-10, MIP1α, TNF-α, IL-6 and IL-8 after 5 h stimulation. OMVs promoted upregulation of all these mediators in Caco-2 cells in the apical compartment (**Figure 3B**). Interestingly, the comparison of data from stimulations performed with vesicles isolated from both strains revealed that OMVs from the probiotic strain EcN promoted a significantly higher increase in the expression of the anti-inflammatory cytokine IL-10 (**Figure 3B**).

Gene expression analysis in PBMCs collected from the basolateral compartment also revealed higher mRNA levels of these mediators than in PBMCs collected from untreated co-cultures (**Figure 3C**). However, due to variability in the data, the results did not reach statistical significance. In this case, the relative increased gene expression values were lower than those of PBMCs directly stimulated by OMVs. These results are consistent with the stimulation of PBMCs by soluble factors released from epithelial cells upon exposure to OMVs.

**FIGURE 4:**
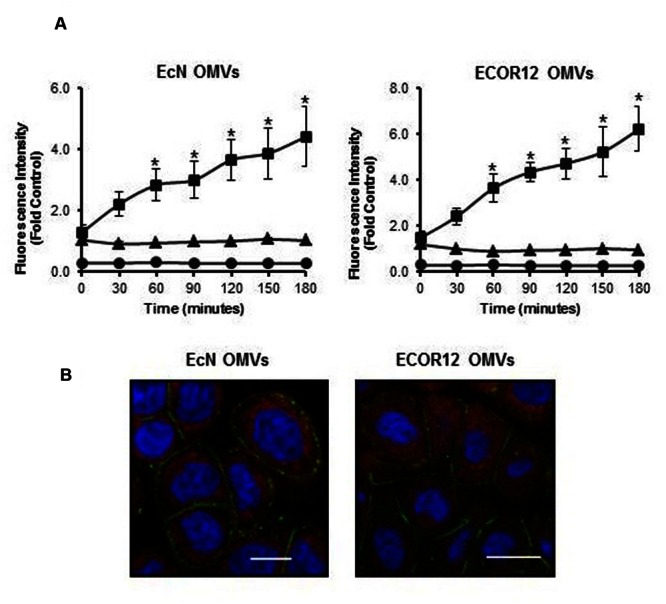
**Outer membrane vesicles uptake by differentiated Caco-2 cells. (A)** Rhodamine B-R18-labeled OMVs from EcN or ECOR12 were applied to the apical side of polarized Caco-2 cells and fluorescence was measured over time (squares). OMVs (triangles) and cells (circles) alone were used as negative controls for background fluorescence. Statistical differences versus the for background fluorescence emitted by OMVs alone were assessed by one-way ANOVA followed by Tukey’s test (^∗^*p* < 0.05). **(B)** Visualization of internalized OMVs by fluorescence microscopy. Caco-2 cells were incubated with rhodamine B-R18 labeled OMVs for 1 h at 37°C. The cell membrane was visualized by immunostaining with antibodies against the zonula occludens ZO-1 protein followed by Alexa Fluor 488-conjugated secondary antibody (green). Nuclei were stained with DAPI (blue). Internalized rhodamine-R18 labeled OMVs are visualized in red. Bar: 20 μm.

In this co-culture system, the different cellular responses to OMVs or to bacterial lysates may be attributed to specific OMV-mediated communication and signaling mechanisms in the epithelial barrier. Thus, we sought to prove that OMVs from these *E. coli* strains are internalized in differentiated Caco-2 cells. We took advantage of the properties of rhodamine isothiocyanate B-R18 fluorescent dye, whose fluorescence is quenched when intercalated into bilayer membranes at a high concentration. However, this dye fluoresces when diluted upon membrane fusion and internalization. Rhodamine B-R18-labeled OMVs applied to the apical side of differentiated Caco-2 cells produced a time-dependent increase in fluorescence. In contrast, no changes in fluorescence emission were observed in non-treated cells or samples containing only labeled-OMVs (**Figure 4A**). These results are compatible with OMVs uptake by intestinal epithelial cells. Internalization of OMVs by Caco-2 cells was assessed by confocal fluorescence microscopy at 1 h incubation with rhodamine B-R18-labeled OMVs. Immunostaining of the peripherally associated membrane protein ZO-1 was performed as an epithelial cell membrane marker. Results presented in **Figure 4B** confirmed the presence of EcN and ECOR12 OMVs in the cytoplasm of Caco-2 cells. Therefore, internalized OMVs can mediate microbiota immunomodulatory effects in the intact intestinal mucosa.

### OMVs from EcN and ECOR12 Modulate the Expression of Immuno-modulatory and Defense Mediators in *Ex Vivo* Colon Explants

**FIGURE 5:**
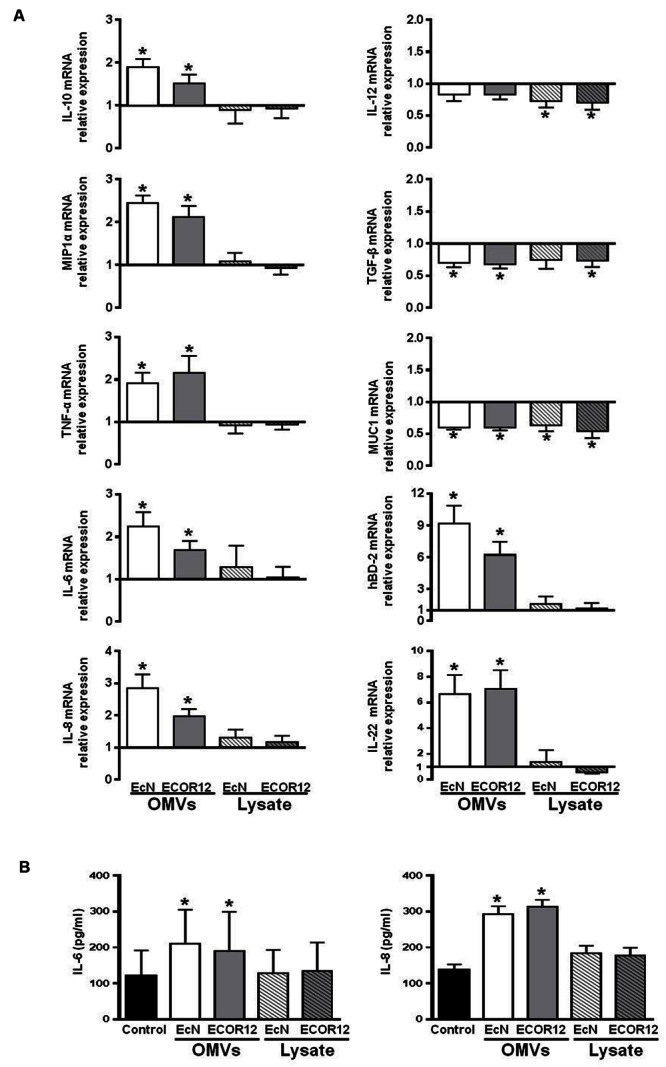
**Expression levels of the indicated immunomodulatory and defense mediators in human colonic explants.** Colonic tissues were challenged for 5 h with OMVs or bacterial lysates from EcN or ECOR12 strains. **(A)** Relative mRNA levels of the indicated mediators. Data are presented as fold-change compared to untreated colon fragments. **(B)** IL-8 and IL-6 protein levels in culture supernatants. Data are presented as mean ± SEM. Statistical differences were assessed by the *t-*test. ^∗^*p* ≤ 0.05, versus control.

In this study, the colon organ culture system was used as a closer model to the *in vivo* conditions. Gene expression levels of the selected mediators were analyzed by RT-qPCR (**Figure 5A**). The expression profile of the cytokines IL-10, MIP1α, TNF-α, IL-6, and IL-8 correlated well with the data obtained in the co-culture system. The corresponding genes were upregulated in colonic explants incubated with OMVs, but not in those incubated with bacterial lysates. After 5 h stimulation, statistically significant increases in the cytokine secreted levels were only observed for IL-6 and IL-8 (**Figure 5B**).

In this model, we also examined the expression of other genes related with host immunomodulatory or defense responses, including IL-12, TGF-β, IL-22, hBD-2, hBD-1, and MUC1. OMVs from either EcN or ECOR12 activated transcription of both IL-22 and the antimicrobial peptide hBD-2, while bacterial lysates failed to do so (**Figure 5A**). In contrast, a different expression profile was seen for the other mediators. Expression of both the proinflammatory cytokine IL-12 and TGF-β was downregulated either by OMVs or bacterial lysates. Expression of MUC1 was also significantly reduced (by nearly 40%) upon treatment with all the bacterial samples (**Figure 5A**). Gene encoding the antimicrobial peptide hBD-1 was not differentially expressed in any of the conditions tested (not shown).

Although, samples from both EcN and ECOR12 strains promoted the same expression profile, colonic explants stimulated with OMVs of the probiotic strain EcN showed a tendency toward better anti-inflammatory balance. The values of IL-10/IL-12 and IL-10/TNF-α mRNA ratios were higher in EcN-treated explants than in ECOR12-treated explants. These values were 2.50 ± 0.28 versus 1.86 ± 0.20 for IL-10/IL-12 and 1.08 ± 0.18 versus 0.80 ± 0.12 for IL-10/TNF-α.

## Discussion

In the gut, communication between microbiota and intestinal mucosa cells has to be established by soluble mediators that can diffuse through the mucin layer. Among the bacterial secreted factors, membrane vesicles have a key role in bacteria-host communication, allowing the delivery of effector molecules upon interaction and internalization into the host cells. Many studies performed in the last decade with pathogens evidenced the role of bacterial membrane vesicles as an important mechanism to deliver virulence and immunomodulatory factors to mammalian target cells. However, reports on microbiota vesicles are still scarce.

We focused our study to assess whether OMVs released by probiotic and commensal *E. coli* strains mediate immune modulation in cellular models that mimic damaged or intact intestinal epithelial barrier. To compare the OMVs activity with that of other bacterial fractions, parallel stimulations were performed with bacterial lysates. We analyzed expression of the chemokine MIP1α, the anti-inflammatory cytokine IL-10, and the pro-inflammatory cytokines IL-6, IL-8, and TNF-α. Our results show that direct stimulation with both EcN and ECOR12 OMVs triggered upregulation of all these mediators in PBMCs. In these cells, the genes regulated by EcN lysates had been identified through microarray analysis (Güttsches et al., 2012). The study showed that upregulation of IL-6, IL-8, and TNF-α by the probiotic strain EcN is mediated either by purified LPS or lysates, whereas upregulation of IL-10 is mainly activated by components of the EcN lysate that are not yet identified (Güttsches et al., 2012). Accordingly, the presence of LPS in *E. coli* OMVs may explain the activation of the expression of IL-6, IL-8, and TNF-α, but upregulation of IL-10 by OMVs may be attributed to another vesicle factor. In this context, it should be stressed that EcN OMVs contain some proteins known to modulate the host immune response, such as the flagellar components FlgE and FlgK, and the cytoplasmic moonlighting proteins glyceraldehyde-3-phosphate dehydrogenase and enolase (Aguilera et al., 2014). These moonlighting proteins can switch between different functions, depending on the cell location. Besides their basic metabolic function, when secreted, these proteins fulfill functions that enable bacteria to colonize and modulate the host immune response. As presented above, cytokine secretion levels were lower in PBMCs exposed to OMVs than in cells exposed to bacterial lysates. The greatest difference was observed for IL-10. For this anti-inflammatory cytokine, the secretion levels were more than 10-fold higher in cells challenged with bacterial lysates, whereas the relative mRNA levels were similar for both OMV and lysate-stimulated cells. According to the molecular composition, we could speculate that bacterial lysates and OMVs activate different signaling pathways or post-transcriptional regulatory mechanisms that would account for the different cytokine secreted levels after 24 h incubation.

The profile of cytokine production by PBMCs incubated with microbiota lysates was clearly different from the profile of cytokine secretion in the co-culture model, in which the epithelial barrier formed by differentiated Caco-2 cells prevent direct access of the effector molecules to underlying PBMCs. In this model, secretion of the analyzed cytokines did not increase over the control levels when the co-culture was apically stimulated with bacterial lysates. Thus, intestinal epithelial cells were poorly responsive to lysate components, which are mainly enriched with cytoplasmic proteins and factors. In contrast, OMVs elicited an immune response in the epithelial cell monolayer as shown by the increased gene expression levels of all the cytokines and chemokines analyzed. This is compatible with the presence in *E. coli* OMVs of PPR-ligands, such as LPS (TLR-4 ligand) or peptidoglycan (NOD-1/NOD-2 ligand) that can trigger NF-kappa B activation. Here, we have shown that microbiota *E. coli* OMVs are internalized in differentiated Caco-2 cells. Therefore, vesicle uptake can assist the delivery of specific bacterial ligands to cytosolic NOD-like receptors. In this co-culture model, activation of the underlying PBMCs upon apical stimulation by microbiota OMVs was assessed by RT-qPCR. Although, the relative gene expression values were not statistically significant after 5 h incubation, the mRNA levels were manifestly higher in co-cultures challenged with OMVs than in non-treated cultures. Upregulation and increased secretion of IL-10, TNF-α, and IL-6 was also described in a co-culture model of intestinal epithelial cells and dendritic cells after apical stimulation with EcN bacterial cells (Zoumpopoulou et al., 2009). Our results prove that PBMCs are stimulated by soluble factors released from epithelial cells upon exposure to OMVs, and show for the first time the ability of microbiota vesicles to mediate signaling events through the intestinal epithelial barrier.

The role of commensal and probiotic *E. coli* OMVs as modulators of the intestinal homeostasis and immunity was corroborated in the *ex vivo* model of colon organ culture, which is closer to the *in vivo* conditions. As in the co-culture model, upregulation of MIP1α, IL-10, TNF-α, IL-6, and IL-8 in colonic mucosa explants was only observed in samples exposed to OMVs. These vesicles also promoted upregulation of the antimicrobial peptide hBD-2. Epithelial hBD-2 plays an important role in intestinal barrier function and many probiotics can induce its secretion (Wehkamp et al., 2004; Möndel et al., 2009). Induction of the expression of hBD-2 by EcN depends on flagella synthesis, as EcN mutants with deletions in genes *fliC* or *flgE* fail to induce this antimicrobial peptide (Schlee et al., 2007). Interestingly, not all *E. coli* strains activate hBD-2 expression (Wehkamp et al., 2004; Möndel et al., 2009). Our results show that OMVs from both EcN and ECOR12 promote hBD-2 upregulation, with higher relative expression levels in colonic fragments challenged with the probiotic OMVs. Induction may be mediated by flagella-associated proteins present in EcN OMVs, such as FliC or FlgE (Aguilera et al., 2014). These results provide evidence that OMVs released by commensal bacteria may also have positive impact on inducible antimicrobial defense mechanisms. In contrast, OMVs from these *E. coli* strains do not significantly modify expression of hBD-1. This antimicrobial peptide is constitutively expressed at high levels by colonic epithelial cells, and functions as a component of the host innate defenses at the mucosal surface. Our results are in accordance with the expression pattern reported for this β-defensin. In contrast to hBD-2, expression of hBD-1 is not inducible by bacterial components or inflammatory stimuli. However, certain enteric pathogens promote hBD-1 downregulation as a mechanism to overcome natural host defenses (reviewed by Cobo and Chadee, 2013).

Another cytokine upregulated in colonic explants by EcN and ECOR12 OMVs is IL-22. This cytokine is mainly expressed by immune cells and displays both pro-inflammatory and anti-inflammatory functions, depending on the tissue and the stimuli that direct its secretion. The pro-inflammatory properties are linked to its production by activated T helper 17 (Th17) cells. The IL-22 targets are typically non-hematopoietic cells, such as epithelial cells. In the intestine, the innate lymphoid cells resident in the lamina propria are a main source of IL-22. This population of cells is essential for the integration of microbiota-derived signals and the control of the adaptive immune response. IL-22 released by gut innate lymphoid cells helps to maintain the integrity of the epithelial barrier by inducing the expression of β-defensins and the production of mucin by globet cells (Nikoopour et al., 2015). Therefore, IL-22 has a relevant role in host-microbiota homeostasis. Expression of this cytokine by innate lymphoid cells and T cells is activated by certain microbiota groups known to protect against food allergen sensitization, such as Clostridia. As a result, increased IL-22 levels reinforce the intestinal epithelial barrier, and therefore limit the access of allergens to the systemic circulation (Stefka et al., 2014). Upregulation of IL-22 by OMVs from EcN and ECOR12 strongly suggest that certain commensal *E. coli* strains can confer intestinal barrier protection, and prevent aberrant responses to food components. In addition, this finding supports the role of microbiota vesicles as a mechanism to deliver regulatory signals to the immune cells resident in the intestinal mucosa.

The expression of genes encoding IL-12, TGF-β and MUC1 was reduced in colonic explants challenged with bacterial samples. Microbiota OMVs and bacterial lysates promoted significant downregulation of TGF-β and MUC1. Relative expression levels were reduced by 25 and 40%, respectively. Expression of the pro-inflammatory cytokine IL-12 was also downregulated, although to a lesser extent.

TGF-β is a pleiotropic cytokine with potent regulatory and inflammatory activities. Its effects depend on cellular and environmental factors. This mediator promotes differentiation of induced Treg cells, which secrete anti-inflammatory cytokines such as IL-10, and help to control inflammation. However, in the presence of IL-6, TGF-β can trigger differentiation of Th17 cells through induction of the transcriptional factor Runx1, promoting further inflammation (Sanjabi et al., 2009; Liu et al., 2015). Th17 cells are especially abundant in the intestinal mucosa surfaces, where, in cooperation with Treg cells, they contribute to preserving intestinal homeostasis. Alteration in the Th17 (pro-inflammatory)/Treg (anti-inflammatory) balance toward excessive production of Th17 cells leads to the pathogenesis of inflammatory bowel diseases (Llopis et al., 2009). Several probiotics favor the Treg response leading to the induction and secretion of anti-inflammatory cytokines such as IL-10 and TGF-β. Our results show that EcN and ECOR12 trigger downregulation of TGF-β expression. As the pro-inflammatory effects of TGF-β are related with the differentiation of Th17 cells, the reduction in TGF-β levels promoted by these microbiota strains may contribute to restoring the Th17/Treg balance under inflammatory conditions. This mechanism may explain the effectiveness of the probiotic EcN in the remission of ulcerative colitis (Kruis et al., 2004).

MUC1 is a membrane-anchored mucin, located in the apical surface of mucosal epithelial cells. Production of this mucin by colonic epithelial cells is stimulated by IL-17 released by Th17 cells, and MUC1 in turn negatively regulates the Th17 cell responses in inflamed gut (Nishida et al., 2012). In fact the MUC1 gene has been linked with susceptibility to inflammatory bowel disease (Apostolopoulos et al., 2015). This mucin has been associated with barrier functions. However, different models have shown contradictory results. Deficiency of MUC1 results in increased colonic permeability and IL-17 responses in knockout mice lacking the T cell receptor (Nishida et al., 2012), whereas knockdown of MUC1 in corneal epithelial cells did not modify the barrier function (Gipson et al., 2014). *In vitro* studies performed in the colon adenocarcinoma cell line LS174T showed upregulation of MUC1 by probiotic strains, including EcN. However, this effect was not observed in an *in vivo* murine model (Becker et al., 2013). Similarly, analysis of MUC1 expression in mice colonized with EcN during the first week of life did not reveal any increase in the MUC1 mRNA levels when compared to the control group (Bergström et al., 2012).

Interestingly, both MUC1 and TGF-β are overexpressed in several cancer types (Khanh do et al., 2013; Apostolopoulos et al., 2015). As EcN and commensal *E. coli* strains promote downregulation of both mediators in intestinal mucosa explants, we may hypothesize that these microbiota strains could help to reduce cancer progression or to increase treatment effectiveness (Viaud et al., 2015). This is especially interesting in the context of immunotherapy strategies in which the individual response to these therapies has been shown to be dependent on gut microbiota (Sivan et al., 2015; Vétizou et al., 2015).

## Conclusion

Recent knowledge supports that microbiota is a source of regulatory signals that influence the development and maturation of the digestive and immune systems. Nowadays, potential clinical applications of gut microbes are foreseen. However, translation of microbiota-based drugs to human health care requires deep knowledge of the molecular mechanisms involved in microbiota-host interaction (Jia et al., 2008; Shanahan, 2011). Our study proves the ability of microbiota vesicles to mediate signaling events to the immune system through the intestinal epithelial barrier. Therefore, OMVs are an effective strategy used by beneficial bacteria of the human microbiota to communicate with intestinal mucosa cells, promoting the delivery of mediators that trigger host immune and defense responses. The *in vivo* beneficial effects of the probiotic EcN on gut homeostasis, especially modulation of the immune response and barrier function, can be mediated by released OMVs.

## Author Contributions

LB, JB, MA, conceived of the study with the participation of EV and RG in experimental design. LB and JB wrote the manuscript. MA, EV, RG, LA, and MJF carried out data interpretation and statistical analysis as well as participated in revising the manuscript. MJF and LA prepared OMVs, Caco-2 cultures, PBMC isolation, Caco-2/PBMC cocultures, cytokine analysis and related work. MJF, LA with the help of EV and MA participated in the e*x vivo* experiments performed with colonic mucosa explants. MAC performed OMVs labeling and internalization studies in Caco-2 cells. All authors read and approved the final manuscript.

## Conflict of Interest Statement

The authors declare that the research was conducted in the absence of any commercial or financial relationships that could be construed as a potential conflict of interest.

## References

[B1] Aguilera L. Toloza L. Giménez R. Odena A. Oliveira E. Aguilar J. (2014). Proteomic analysis of outer membrane vesicles from the probiotic strain *Escherichia coli* Nissle 1917. *Proteomics* 14 222–229. 10.1002/pmic.201300328 24307187

[B2] Apostolopoulos V. Stojanovska L. Gargosky S. E. (2015). MUC1 (CD227): a multi-tasked molecule. *Cell. Mol. Life Sci.* 72 4475–4500. 10.1007/s00018-015-2014-z 26294353 PMC11113675

[B3] Arribas B. Rodríguez-Cabezas M. E. Camuesco D. Comalada M. Bailón E. Utrilla P. (2009). A probiotic strain of *Escherichia coli*, Nissle 1917 given orally exerts local and systemic anti-inflammatory effects in lipopolysaccharide-induced sepsis in mice. *Br. J. Pharmacol.* 157 1024–1033. 10.1111/j.1476-5381.2009.00270.x 19486007 PMC2737661

[B4] Becker S. Oelschlaeger T. A. Wullaert A. Vlantis K. Pasparakis M. Wehkamp J. (2013). Bacteria regulate intestinal epithelial cell differentiation factors both in vitro and in vivo. *PLoS ONE* 8:e55620 10.1371/journal.pone.0055620 PMC3572096 23418447

[B5] Belkaid Y. Hand T. W. (2014). Role of the microbiota in immunity and inflammation. *Cell* 157 121–141. 10.1016/j.cell.2014.03.011 24679531 PMC4056765

[B6] Bergström A. Kristensen M. B. Bahl M. I. Metzdorff S. B. Fink L. N. Frøkiaer H. (2012). Nature of bacterial colonization influences transcription of mucin genes in mice during the first week of life. *BMC Res. Notes* 5:402 10.1186/1756-0500-5-402 PMC3465226 22857743

[B7] Bomberger J. M. Maceachran D. P. Coutermarsh B. A. Ye S. O’Toole G. A. Stanton B. A. (2009). Long-distance delivery of bacterial virulence factors by *Pseudomonas aeruginosa* outer membrane vesicles. *PLoS Pathog.* 5:e1000382 10.1371/journal.ppat.1000382 PMC2661024 19360133

[B8] Borruel N. Casellas F. Antolin M. Llopis M. Carol M. Espíin E. (2003). Effects of nonpathogenic bacteria on cytokine secretion by human intestinal mucosa. *Am. J. Gastroenterol.* 98 865–870. 10.1111/j.1572-0241.2003.07384.x 12738469

[B9] Caballero S. Pamer E. G. (2015). Microbiota-mediated inflammation and antimicrobial defense in the intestine. *Annu. Rev. Immunol.* 33 227–256. 10.1146/annurev-immunol-032713-120238 25581310 PMC4540477

[B10] Chatterjee D. Chaudhuri K. (2013). Vibrio cholerae O395 outer membrane vesicles modulate intestinal epithelial cells in a NOD1 protein-dependent manner and induce dendrític cell-mediated Th2/Th17 cell responses. *J. Biol. Chem.* 288 4299–4309. 10.1074/jbc.M112.408302 23275338 PMC3567681

[B11] Chibbar R. Dieleman L. A. (2015). Probiotics in the management of ulcerative colitis. *J. Clin. Gastroenterol.* 49 S50–S55. 10.1097/MCG.0000000000000368 26447965

[B12] Cobo E. R. Chadee K. (2013). Antimicrobial human b-defensins in the colon and their role in infectious and non-infectious diseases. *Pathogens* 2 177–192. 10.3390/pathogens2010177 25436887 PMC4235710

[B13] Egea L. Aguilera L. Giménez R. Sorolla M. A. Aguilar J. Badía J. (2007). Role of secreted glyceraldehyde-3-phosphate dehydrogenase in the infection mechanism of enterohemorrhagic and enteropathogenic *Escherichia coli*: interaction of the extracellular enzyme with human plasminogen and fibrinogen. *Int. J. Biochem. Cell Biol.* 39 1190–1203. 10.1016/j.biocel.2007.03.008 17449317

[B14] Ellis T. N. Kuehn M. J. (2010). Virulence and immunomodulatory roles of bacterial outer membrane vesicles. *Microbiol. Mol. Biol. Rev.* 74 81–94. 10.1128/MMBR.00031-09 20197500 PMC2832350

[B15] Fang H. W. Fang S. B. Chiang Chiau J. S. Yeung C. Y. Chan W. T. Jiang C. B. (2010). Inhibitory effects of *Lactobacillus casei* subsp. rhamnosus on *Salmonella* lipopolysaccharide-induced inflammation and epithelial barrier dysfunction in a co-culture model using Caco-2/peripheral blood mononuclear cells. *J. Med. Microbiol.* 59 573–579. 10.1099/jmm.0.009662-0 20110387

[B16] Foxx-Orenstein A. E. Chey W. D. (2012). Manipulation of the gut microbiota as a novel treatment strategy for gastrointestinal disorders. *Am. J. Gastroenterol. Suppl.* 1 41–46. 10.1038/ajgsup.2012.8

[B17] Garrett W. S. Gordon J. I. Glimcher L. H. (2010). Homeostasis and inflammation in the intestine. *Cell* 140 859–870. 10.1016/j.al.2010.01.02 20303876 PMC2845719

[B18] Gipson I. K. Spurr-Michaud S. Tisdale A. Menon B. B. (2014). Comparison of the transmembrane mucins MUC1 and MUC16 in epithelial barrier function. *PLoS ONE* 9:e100393 10.1371/journal.pone.0100393 PMC4072602 24968021

[B19] Güttsches A. K. Löseke S. Zähringer U. Sonnenborn U. Enders C. Gatermann S. (2012). Anti-inflammatory modulation of immune response by probiotic *Escherichia Coli* Nissle 1917 in human blood mononuclear cells. *Innate Immun.* 18 204–216. 10.1177/1753425910396251 21382908

[B20] Hafez M. Hayes K. Goldrick M. Grencis R. K. Roberts I. S. (2010). The K5 capsule of *Escherichia coli* strain Nissle 1917 is important in stimulating expression of Toll-like receptor 5, CD14, MyD88, and TRIF together with the induction of interleukin-8 expression via the mitogen-activated protein kinase pathway in epithelial cells. *Infect. Immun.* 78 2153–2162. 10.1128/IAI.01406-09 20145095 PMC2863526

[B21] Haller D. Bode C. Hammes W. P. Pfeifer A. M. Schiffrin E. J. Blum S. (2000). Non-pathogenic bacteria elicit a differential cytokine response by intestinal epithelial cell/leucocyte co-cultures. *Gut* 47 79–87. 10.1136/gut.47.1.79 10861268 PMC1727962

[B22] Jacobs J. P. Braun J. (2014). Immune and genetic gardening of the intestinal microbiome. *FEBS Lett.* 588 4102–4111. 10.1016/jfehslet.2014.02.052 24613921 PMC4156569

[B23] Jia W. Li H. Zhao L. Nicholson J. K. (2008). Gut microbiota: a potential new territory for drug targeting. *Nat. Rev. Drug Discov.* 7 123–129. 10.1038/nrd2505 18239669

[B24] Johansson M. E Phillipson M. Petersson J. Velcich A. Holm L. Hansson G. C. (2008). The inner of the two Muc2 mucin-dependent mucus layers in colon is devoid of bacteria. *Proc. Natl. Acad. Sci. U.S.A.* 105 15064–15069. 10.1073/pnas.0803124105 18806221 PMC2567493

[B25] Kang C. S. Ban M. Choi E. J. Moon H. G. Jeon J. S. Kim D. K. (2013). Extracellular vesicles derived from gut microbiota, especially *Akkermansia muciniphila*, protect the progression of dextran sulfate sodium-induced colitis. *PLoS ONE* 8:e76520 10.1371/journal.pone.0076520 PMC3811976 24204633

[B26] Kaparakis-Liaskos M. Ferrero R. L. (2015). Immune modulation by bacterial outer membrane vesicles. *Nat. Rev. Immunol.* 15 375–387. 10.1038/nri3837 25976515

[B27] Khanh do T. Mekata E. Mukaisho K. Sugihara H. Shimizu T. Shiomi H. (2013). Transmembrane mucin MUC1 overexpression and its association with CD10+ myeloid cells, transforming growth factor-β1 expression, and tumor budding grade in colorectal cancer. *Cancer Sci.* 104 958–964. 10.1111/cas.12170 23566254 PMC7657147

[B28] Kim J. H. Lee J. Park J. Gho Y. S. (2015). Gram-negative and Gram-positive bacterial extracellular vesicles. *Semin. Cell Dev. Biol.* 40 97–104. 10.1016/j.semcdb.2015.02.006 25704309

[B29] Krishnan S. Alden N. Lee K. (2015). Pathways and funcions of gut microbiota metabolism impacting host physiology. *Curr. Opin. Biotechnol.* 36 137–145. 10.1016/j.cell.2014.03.011 26340103 PMC4688195

[B30] Kruis W. Fric P. Pokrotnieks J. Lukás M. Fixa B. Kascák M. (2004). Maintaining remission of ulcerative colitis with the probiotic *Escherichia coli* Nissle 1917 is as effective as with standard mesalazine. *Gut* 53 1617–1623. 10.1136/gut.2003.037747 15479682 PMC1774300

[B31] Kulp A. Kuehn M. J. (2010). Biological functions and biogenesis of secreted bacterial outer membrane vesicles. *Annu. Rev. Microbiol.* 64 163–184. 10.1146/annurev.micro.091208.073413 20825345 PMC3525469

[B32] Le Chatelier E. Nielsen T. Qin J. Prifti E. Hildebrand F. Falony G. (2013). Richness of human gut microbiome correlates with metabolic markers. *Nature* 500 541–546. 10.1038/nature12506 23985870

[B33] Liu H. P. Cao A. T. Feng T. Li Q. Zhang W. Yao S. (2015). TGF-β converts Th1 cells into Th17 cells trough stimulation of Runx1 expression. *Eur. J. Immunol.* 45 1010–1018. 10.1002/eji.201444726 25605286 PMC4441226

[B34] Llopis M. Antolin M. Carol M. Borruel N. Casellas F. Martinez C. (2009). Lactobacillus casei downregulates commensals’ inflammatory signals in Crohn’s disease mucosa. *Inflamm. Bowel Dis.* 15 275–283. 10.1002/ibd.20736 18839424

[B35] López P. González-Rodríguez I. Sánchez B. Gueimonde M. Margolles A. Suárez A. (2012). Treg-inducing membrane vesicles from *Bifidobacterium bifidum* LMG13195 as potential adjuvants in immunotherapy. *Vaccine* 30 825–829. 10.1016/j.vaccine.2011.11.115 22172507

[B36] Lowry O. H. Rosebrough N. J. Farr A. L. Randall R. J. (1951). Protein measurement with the Folin phenol reagent. *J. Biol. Chem.* 193 265–273. 14907713

[B37] Möndel M. Schroeder B. O. Zimmermann K. Huber H. Nuding S. Beisner J. (2009). Probiotic *E. coli* treatment mediates antimicrobial human beta-defensin synthesis and fecal excretion in humans. *Mucosal Immunol.* 2 166–172. 10.1038/mi.2008.77 19129752 PMC10026704

[B38] Nikoopour E. Bellemore S. M. Singh B. (2015). IL-22, cell regeneration and autoimmunity. *Cytokine* 74 35–42. 10.1016/j.cyto.2014.09.007 25467639

[B39] Nishida A. Lau C. W. Zhang M. Andoh A. Shi H. N. Mizoguchi E. (2012). The membrane-bound mucin Muc1 regulates T helper 17-cell responses and colitis in mice. *Gastroenterology* 142 865–874. 10.1053/j.gastro.2011.12.036 22202458 PMC3441148

[B40] O’Hara A. M. Shanahan F. (2006). The gut flora as a forgotten organ. *EMBO Rep.* 7 688–693. 10.1038/sj.embor.7400731 16819463 PMC1500832

[B41] Ochman H. Selander R. K. (1984). Standard reference strains of *Escherichia coli* from natural populations. *J. Bacteriol.* 157 690–693. 6363394 10.1128/jb.157.2.690-693.1984 PMC215307

[B42] Olsen I. Amano A. (2015). Outer membrane vesicles-offensive weapons or good Samaritans? *J*. *Oral Microbiol.* 7 27468 10.3402/jom.v7.27468 PMC4385126 25840612

[B43] Pozo-Rubio T. Mujico J. R. Marcos A. Puertollano E. Nadal I. Sanz Y. (2011). Immunostimulatory effect of faecal *Bifidobacterium* species of breast-fed and formula-fed infants in a peripheral blood mononuclear cell/Caco-2 co-culture system. *Br. J. Nutr.* 106 1216–1223. 10.1017/S0007114511001656 21736809

[B44] Robles-Alonso V. Guarner F. (2013). Linking the gut microbiota to human health. *Br. J. Nutr.* 2 S21–S26. 10.1017/S0007114512005235 23360877

[B45] Sanjabi S. Zenewicz L. A. Kamanaka M. Flavell R. A. (2009). Anti-inflammatory and pro-inflammatory roles of TGF-beta, IL-10, and IL-22 in immunity and autoimmunity. *Curr. Opin. Pharmacol.* 9 447–453. 10.1016/j.coph.2009.04.008 19481975 PMC2755239

[B46] Schaar V. de Vries S. P. Perez Vidakovics M. L. Bootsma H. J. Larsson L. Hermans P. W. (2011). Multicomponent *Moraxella catarrhalis* outer membrane vesicles induce an inflammatory response and are internalized by human epithelial cells. *Cell. Microbiol.* 13 432–449. 10.1111/j.1462-5822.2010.01546.x 21044239

[B47] Schertzer J. W. Whiteley M. (2013). Bacterial outer membrane vesicles in trafficking, communication and the host-pathogen interaction. *J. Mol. Microbiol. Biotechnol.* 23 118–130. 10.1159/000346770 23615200

[B48] Schlee M. Wehkamp J. Altenhoefer A. Oelschlaeger T. A. Stange E. F. Fellermann K. (2007). Induction of human beta-defensin 2 by the probiotic *Escherichia coli* Nissle 1917 is mediated through flagellin. *Infect. Immun.* 75 2399–2407. 10.1128/IAI.01563-06 17283097 PMC1865783

[B49] Sears C. L. Garrett W. S. (2014). Microbes, microbiota and colon cancer. *Cell Host Microbe* 15 317–328. 10.1016/j.chom.2014.02.007 24629338 PMC4003880

[B50] Shanahan F. (2011). The gut microbiota in 2011: translating the microbiota to medicine. *Nat. Rev. Gastroenterol. Hepatol.* 9 72–74. 10.1038/nrgastro.2011.250 22183186

[B51] Shen Y. Giardino Torchia M. L. Lawson G. W. Karp C. L. Ashwell J. D. Mazmanian S. K. (2012). Outer membrane vesicles of a human commensal mediate immune regulation and disease protection. *Cell Host Microbe* 12 509–520. 10.1016/j.chrom.2012.08.004 22999859 PMC3895402

[B52] Sivan A. Corrales L. Hubert N. Williams J. B. Aquino-Michaels K. Earley Z. M. (2015). Commensal *Bifidobacterium* promotes antitumor immunity and facilitates anti-PD-L1 efficacy. *Science* 350 1084–1089. 10.1126/science.aac4255 26541606 PMC4873287

[B53] Stefka A. T. Feehley T. Tripathi P Qiu J. McCoy K. Mazmanian S. K. (2014). Commensal bacteria protect against food allergen sensitization. *Proc. Natl. Acad. Sci. U.S.A.* 111 13145–13150. 10.1073/pnas.1412008111 25157157 PMC4246970

[B54] Stentz R. Horn N. Cross K. Salt L. Brearley C. Livermore D. M. (2015). Cephalosporinases associated with outer membrane vesicles released by *Bacteroides* spp. protect gut pathogens and commensals against β-lactam antibiotics. *J. Antimicrob. Chemother.* 70 701–709. 10.1093/jac/dku466 25433011 PMC4319488

[B55] Sturm A. Rilling K. Baumgart D. C. Gargas K. Abou-Ghazalé T. Raupach B. (2005). *Escherichia coli* Nissle 1917 distinctively modulates T-cell cycling and expansion via toll-like receptor 2 signaling. *Infect. Immun.* 73 1452–1465. 10.1128/IAI.73.3.1452-1465.2005 15731043 PMC1064918

[B56] Thaiss C. A. Levy M. Suez J. Elinav E. (2014). The interplay between the innate inmune system and the microbiota. *Curr. Opin. Immunol.* 26 41–48. 10.1016/jcoi.2013.10.016 24556399

[B57] Ukena S. N. Singh A. Dringenberg U. Engelhardt R. Seidler U. Hansen W. (2007). Probiotic *Escherichia coli* Nissle 1917 inhibits leaky gut by enhancing mucosal integrity. *PLoS ONE* 2:e1308 10.1371/journal.pone.0001308 PMC2110898 18074031

[B58] Vaishnava S. Yamamoto M. Severson K. M. Ruhn K. A. Yu X. Koren O. (2011). The antibacterial lectin RegIIIgamma promotes the spatial segregation of microbiota and host in the intestine. *Science* 334 255–258. 10.1126/science.1209791 21998396 PMC3321924

[B59] Vétizou M. Pitt J. M. Daillère R. Lepage P. Waldschmitt N. Flament C. (2015). Anticancer immunotherapy by CTLA-4 blockade relies on the gut microbiota. *Science* 350 1079–1084. 10.1126/science.aad1329 26541610 PMC4721659

[B60] Viaud S. Daillère R. Boneca I. G. Lepage P. Langella P. Chamaillard M. (2015). Gut microbiome and anticancer immune response: really hot Sh*t! *Cell Death Differ*. 22 199–214. 10.1038/cdd.2014.56 24832470 PMC4291500

[B61] Wehkamp J. Harder J. Wehkamp K. Wehkamp-von Meissner B. Schlee M. Enders C. (2004). NF-kappaB- and AP-1-mediated induction of human beta defensin-2 in intestinal epithelial cells by *Escherichia coli* Nissle 1917: a novel effect of a probiotic bacterium. *Infect. Immun.* 72 5750–5758. 10.1128/IAI.72.10.5750-5758.2004 15385474 PMC517557

[B62] Zoumpopoulou G. Tsakalidou E. Dewulf J. Pot B. Grangette C. (2009). Differential crosstalk between epithelial cells, dendritic cells and bacteria in a co-culture model. *Int. J. Food Microbiol.* 131 40–51. 10.1016/j.ijfoodmicro.2008.12.037 19264370

[B63] Zyrek A. A. Cichon C. Helms S. Enders C. Sonnenborn U. Schmidt M. A. (2007). Molecular mechanisms underlying the probiotic effects of *Escherichia coli* Nissle 1917 involve ZO-2 and PKCzeta redistribution resulting in tight junction and epithelial barrier repair. *Cell. Microbiol.* 9 804–816. 10.1111/j.1462-5822.2006.00836.x 17087734

